# CNAttention: an attention-based deep multiple-instance method for uncovering copy number aberration signatures across cancers

**DOI:** 10.1093/bib/bbaf696

**Published:** 2026-01-15

**Authors:** Ziying Yang, Michael Baudis

**Affiliations:** Department of Molecular Life Sciences, University of Zurich, Winterthurerstr. 190, Zurich, CH-8057 Zurich, Switzerland; Swiss Institute of Bioinformatics, Winterthurerstr. 190, CH-8057 Zurich, Switzerland; Department of Molecular Life Sciences, University of Zurich, Winterthurerstr. 190, Zurich, CH-8057 Zurich, Switzerland; Swiss Institute of Bioinformatics, Winterthurerstr. 190, CH-8057 Zurich, Switzerland

**Keywords:** copy number aberrations, cancer classification, cancer heterogeneity, attention-based multiple instance deep learning

## Abstract

Somatic copy number aberrations (CNAs) represent a distinct class of genomic mutations associated with oncogenetic effects. Over the past three decades, significant volumes of CNA data have been generated through molecular-cytogenetic and genome sequencing-based techniques. These data have been pivotal in identifying cancer-related genes and advancing research on the relationship between CNAs and histopathologically defined cancer types. However, comprehensive studies of CNA landscapes and disease parameters are challenging due to the vast diagnostic and genomic heterogeneity encountered in ”pan-cancer” approaches. In this study, we introduce *CNAttention*, an attention-based deep multiple instance learning method designed to comprehensively analyze CNAs across different cancers and uncover specific CNA patterns within integrated gene-level CNA profiles of 30 cancer types. CNAttention effectively learns CNA features unique to each cancer type and generates CNA signatures for 30 cancer types using attention mechanisms, highlighting the distinctiveness of their CNA landscapes. CNAttention demonstrates high accuracy and exhibits stable performance even with the incorporation of external datasets or parameter adjustments, underscoring its effectiveness in tumor identification. Expanding these signatures to cancer classification trees reveals common patterns not only among physiologically related cancer types but also among clinico-pathologically distant types, such as different cancers originating from neural crest derived cells. Additionally, detected signatures also uncover genomic heterogeneity in individual cancer types, for instance in brain lower grade glioma. Additional experiments with classification models underscore the efficacy of these signatures in representing various cancer types and their potential utility in clinical diagnosis.

## Introduction

Copy number variations (CNVs) refer to changes in the number of copies of specific DNA segments in the genome, usually including deletions or duplications with sizes ranging from 1kp to multiple megabases [1]. Several somatic variant discovery tools have been developed to detect CNVs or structural variations from genomic sequencing data, such as GISTIC2.0 [2], FACETS [3], and PRESM [4], which form the foundation for downstream CNV-based analyses. Copy number aberrations (CNAs) refer to acquired CNVs in the disease genome in comparison with the healthy genome, potentially altering the diploid status of specific genomic loci. For instance, neurodevelopmental disorders like intellectual disability, autism, and schizophrenia have been found to be associated with CNAs, accounting for at least 15% of these conditions [5]. The relevance between CNAs and neurodevelopmental diseases may stem from the disruption of gene pathways involved in neuron development. Additionally, several genes implicated in neurodevelopment, such as A2BP1, IMMP2L, and AUTS2, have been reported to harbor mutational CNAs [6].

Cancer initiation and progression are closely linked to changes in copy number [7]. In breast cancer, for instance, Li *et al*. [8] conducted an oncogenetic tree analysis of CNAs and found that the genetic alteration of ErbB2 occurs early, while CNAs of AKT2, PTEN, CCND1, RAS, and PIK3CA are late events. This association can be partially attributed to cellular stress, as copy number changes often occur in response to stressors like hypoxia, which may switch DNA repair mechanisms from homologous recombination to nonhomologous repair [9]. There is ample evidence suggesting that individuals with certain CNAs may be predisposed to cancer [10]. Despite this, the majority of studies have focused on identifying associations with cancer-driver genes or the impact of focal regions in specific tumor types. Consequently, CNA patterns have often been characterized by the coverage of driver genes, rather than through comparative analyses of the entire genome. However, this approach has two drawbacks. Firstly, the distribution of cancer driver genes is highly skewed, with only a few hallmark drivers accounting for a large percentage of tumorigenesis, leaving a long-tail of rare or putative drivers to account for the rest [11]. Secondly, research has revealed various facets of CNAs in relation to cellular regulations and genome dynamics [12–15]. Therefore, CNA patterns that solely rely on driver genes often fail to capture the full spectrum of aberrations. To address this limitation, it would be more comprehensive to abstract CNA patterns based on their characteristic aberrations, rather than focusing solely on focal regions overlapping with driver genes.

While on a global scale, the associations between CNAs and different types of cancer still remain elusive. Some CNAs overlapped between tumor types, others were tumor type-specific; losses of CDH20 and PTEN were observed in both tumor types, whereas amplifications seemed more tumor type-specific, such as EGFR and MAP2K4 in colon cancer and ERBB2 in breast cancer [16]. Some previous studies have been able to delineate diverging patterns in clinically related entities. For example, it could be shown that the CNA patterns between lung adenocarcinoma and squamous cell carcinoma are very different [17] and that there are specific landscapes of mutations and copy number changes in various cancers [18]. Interestingly, cancer type-specific CNAs derived from cell-free DNA (cfDNA) have demonstrated their potential in identifying cancer types and tissues of origin [19–21]. As an emerging field, the accurate identification of genomic abnormalities and classifications of the cfDNA remains challenging, and therefore, the characterization of specific CNA patterns related to cancer types could provide valuable information for such applications.

In this study, we assembled a collection of 10 628 CNA profiles and introduced a novel attention-based method CNAttention, which by capturing specific weighted features of each cancer type in our analysis achieved a classification accuracy of 0.89. When testing our method on additional datasets the accuracy did not deteriorate, showcasing the effectiveness of CNAttention in extracting relevant CNA characteristics of different cancers and in identifying tumors types. Additionally, we used the weights assigned by the attention mechanism to generate specific CNA signatures for 30 cancer types and compared these signatures with the original data and indicators for their biological significance. The result illustrates the genetic uniqueness and relationships of extracted CNA patterns in different cancer types but also uncovers heterogeneity within cancer classifications, therefore demonstrating the potential of CNA signatures in improving diagnostic assessments in oncology.

## Methodology

**Figure 1:**
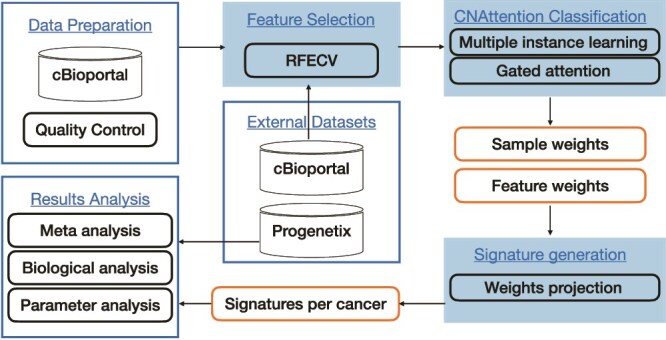
The diagram visualizes the workflow for analyzing cancer CNAs, including data preparation and quality control, feature selection using RFECV to handle high-dimensional data and the CNAttention classification stage which generates interpretable, cancer-type-specific CNA signatures by projecting the model’s attention weights onto genomic features.

As shown in Fig. 1, CNAttention is a three-stage method designed to tackle major challenges in cancer CNA analysis, including data dimensionality, cancer heterogeneity, and model interpretability. The method integrates feature selection with multiple-instance learning (MIL) and attention mechanisms to uncover cancer-specific CNA patterns and generate robust, interpretable CNA signatures.

First, Recursive Feature Elimination with Cross-Validation (RFECV) selects the most informative features, helping reduce overfitting and computation time given the high dimensionality of gene-level CNA profiles. Next, by framing classification as a MIL problem and incorporating attention, we address intra-type variability in cancer samples—focusing the model on representative instances within each bag. Finally, attention-derived instance weights are projected to features to produce CNA signatures per cancer type, reflecting biologically relevant and discriminative patterns.

### Feature selection by Recursive Feature Elimination with Cross-Validation

RFECV iteratively removes the least relevant features based on cross-validation scores, identifying an optimal subset for classification. This reduces noise and the risk of overfitting, especially important in CNA data where features (genes) greatly outnumber samples.


(1) \begin{align*}& \underset{\mathrm{features}}{\mathrm{argmax}} \left( \mathrm{mean}(\mathrm{cross}{\_}\mathrm{val}{\_}\mathrm{score}(\mathrm{est}, X_{\mathrm{features}}, y, \mathrm{scoring})) \right),\end{align*}

where $X$ is the feature matrix, $y$ the target, and est and scoring define the model and metric. This step preserves only biologically relevant CNA signals across cancers.

### Cancer classification by CNAttention

Due to cancer heterogeneity, CNA profiles from the same cancer type may vary significantly. To handle cancer heterogeneity, we formulate the classification task as an MIL problem [22, 23]. In our formulation, each instance corresponds to a single patient’s gene-level CNA profile, while each bag represents a collection of patient instances, carrying one shared label for that cancer type. This setup enables the model to learn generalized CNA patterns that characterize a cancer type rather than fitting individual samples.

Each instance (sample) is treated as unlabeled within a bag, and the bag label reflects the dominant cancer type. Identifying which instances drive the bag label is key; these “key instances” represent the most typical CNA patterns of a cancer class [24]. We extend the attention-based MIL model of [25] to multi-class classification, using neural networks to learn both instance embeddings and class probabilities.

We model the bag label distribution using a multinomial log-likelihood function. This avoids the limitations of max-based MIL (e.g. vanishing gradients) and enables end-to-end training. Three steps are involved: (i) transform instances to embeddings, (ii) aggregate via a symmetric function, and (iii) map to bag probabilities. Each transformation is learned via neural networks, increasing flexibility and expressiveness.


(2) \begin{align*}& Y = \max_{k} y_{k} \quad \text{(assumption for MIL)}\end{align*}

### Multiple-instance learning with neural networks

Let $f_\psi $ be a neural network mapping input $\mathbf{x}_{k}$ to an embedding $\mathbf{h}_{k} = f_\psi (\mathbf{x}_{k})$. These embeddings are input to a bag-level function $g_\phi $ that estimates class probabilities. Parameterizing both $f$ and $g$ with neural networks enables modeling complex, nonlinear dependencies and ensures differentiability for training.

MIL pooling must be permutation-invariant and adaptable. Compared with fixed pooling (e.g. max, mean), attention-based pooling dynamically focuses on informative instances, crucial for capturing diverse CNA manifestations across patients.

### Attention-based multiple-instance learning pooling

We adopt an attention-based pooling mechanism, where each instance contributes to the bag embedding $\mathbf{z}$ based on its learned relevance:


(3) \begin{align*}& \mathbf{z} = \sum_{k=1}^{K} a_{k} \mathbf{h}_{k}\end{align*}

Attention weights $a_{k}$ are computed using a trainable gating mechanism:


(4) \begin{align*}& a_{k} = \frac{\exp\left\{ \mathbf{w}^\top \left[ \tanh(\mathbf{V} \mathbf{h}_{k}^\top) \odot \sigma(\mathbf{U} \mathbf{h}_{k}^\top) \right] \right\}}{\sum_{j=1}^{K} \exp\left\{ \mathbf{w}^\top \left[ \tanh(\mathbf{V} \mathbf{h}_{j}^\top) \odot \sigma(\mathbf{U} \mathbf{h}_{j}^\top) \right] \right\}}\end{align*}

This gated attention mechanism increases model expressiveness and interpretability. It not only improves classification but also identifies which instances most influence the prediction—an essential feature for understanding cancer-specific CNA patterns.

### Signature generation

After training, each instance within a bag (cancer type) has an attention weight $w_{i}$ and an instance-level class probability vector $p_{i}$. We compute the weighted class score for each gene feature by linearly combining the instance-level predictions using their attention weights:


\begin{align*} & \hat{p}_{i,k} = \frac{w_{i,j} \cdot p_{i,k}}{\sum_{j} w_{i,j}},\end{align*}

where $\hat{p}_{g,k}$ represents the aggregated contribution of gene $g$ to class $k$ (cancer type). These normalized per-gene scores are then averaged across all instances of the same cancer type to form the final CNA signature vector for that cancer. Once we have the normalized predictions for each instance, we can determine the predicted class for each instance by selecting the class with the highest normalized prediction. Let $\hat{c}_{i}$ represent the predicted class for instance $i$, which is obtained by


\begin{align*} & \hat{c}_{i} = \arg\max_{k} \hat{p}_{i,k} \end{align*}

We then evaluate instance accuracy as the fraction of correctly predicted samples. Finally, attention-weighted instance predictions are projected to feature weights, generating CNA signatures for each cancer type. These signatures are compact, class-specific, and biologically interpretable, highlighting the most informative CNA regions across the cancer landscape.

CNAttention not only classifies cancer types but also produces compact, interpretable CNA signatures. These signatures reflect the learned patterns underlying classification and can be validated biologically (e.g. via pathway analysis) or against external resources such as Progenetix. The attention mechanism enables clear attribution of predictive weight to key instances and features. Further formulation and derivations are available in Supplementary Methods.

## Results

### Datasets

Gene-level CNA profiles across various cancers were obtained from cBioPortal. CNV values were discretized into five categories: ”-2” (deep loss, likely homozygous deletion), ”-1” (shallow loss, likely heterozygous deletion), ”0” (diploid), ”1” (low-level gain), and ”2” (high-level amplification).

Gene-level CNA profiles of the 30 cancer types (with samples over 50) were provided in the cBioPortal database. The 30 cancer types and the sample numbers are listed in Supplementary Table S1. After gene feature alignments, there are 10 628 CNA profiles on 24 919 genes. The dataset was randomly divided into 80% training and 20% testing subsets. RFECV-based feature selection was performed on the training subset, reducing the number of gene features to 2917. These features were then used in the subsequent attention-based multiple instance learning framework for cancer classification and signature generation. To validate the signatures externally, we projected them onto Progenetix [26–28], a comprehensive reference database for CNV profiles.

### Classification performance analysis

**Figure 2:**
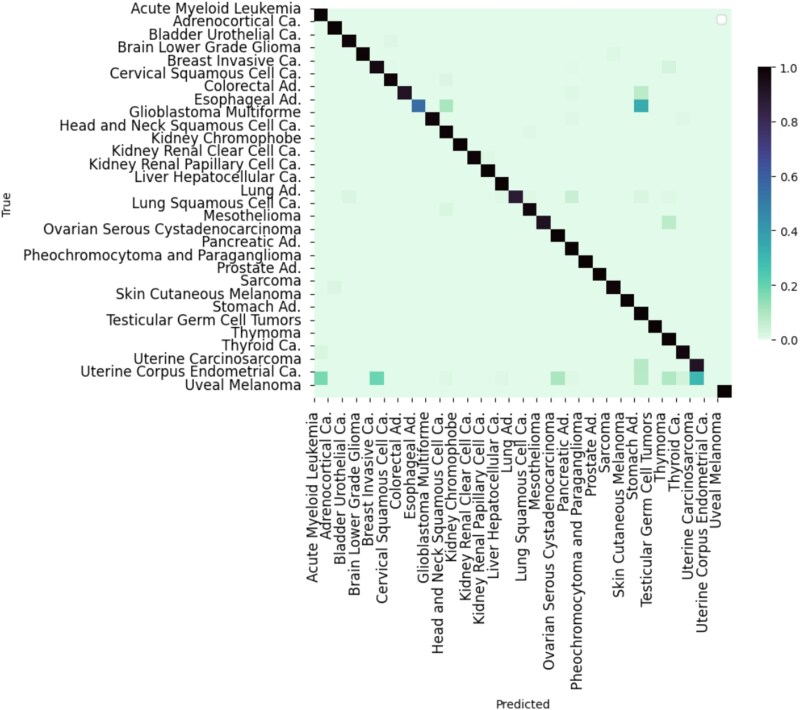
The cancer classification performance of CNAttention. The values in the cells indicate the percentage of biosamples of the corresponding row cancer type classified to the corresponding column cancer type.

**Figure 3:**
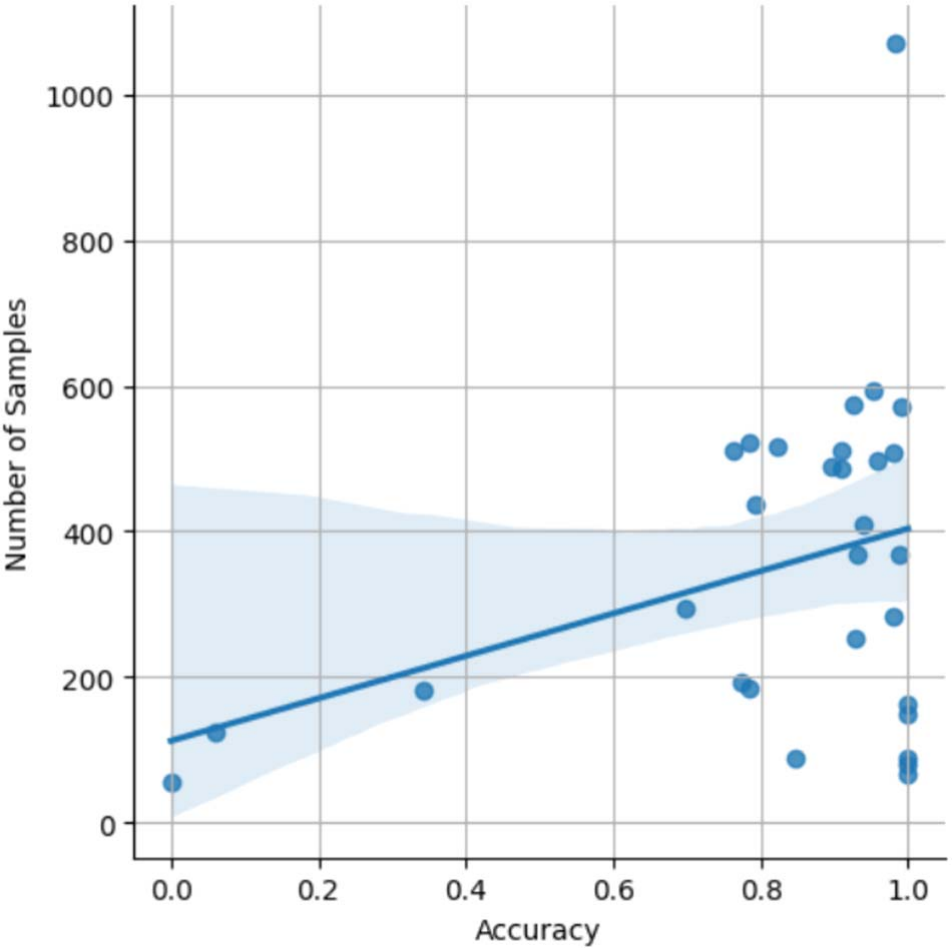
Correlation between classification accuracy and the number of samples.

As shown in Fig. 2, the accuracy for cancer type assignment of samples is high, with an average of 0.89. Exceptions are the misclassification of Uterine Carcinosarcoma and Uterine Corpus Endometrial Carcinoma, and Thymoma and Thyroid Carcinoma. A potential reason can be found in Fig. 3 where the entities with the lowest scores all fall into the area with the lowest sample numbers (below 200). However, for a larger number of entities with <200 samples, classification accuracy remains sufficient, and additional factors such as incorrect diagnostic classification or genomic heterogeneity in those entities might play a role here. For benchmarking of the methodology we compared CNAttention with other methods with and without our feature selection, as shown in Table 1, our method outperforms others. We need to note that comparing with the CNAttention without attention mechanism indicates the effectiveness of the attention mechanism in capturing the CNA patterns of different cancer types. Also, the increase in accuracy of random forest indicates not only the decrease in time costs but also the performance improvement. In addition, we added two external datasets of glioblastoma [29] and colorectal adenocarcinoma [30] for testing; results show that the accuracy remains stable, which proves the robustness of CNAttention.

**Table 1:** Classification comparison with other methods

Method	Average accuracy
**CNAttention**	**0.89**
Random Forest	0.64
Random Forest (with feature selection)	0.65
Zhang *et al*. [31]	0.72
Qiu *et al*. [32]	0.67
CNAttention without attention	0.65

### Copy number aberration signatures

**Figure 4:**
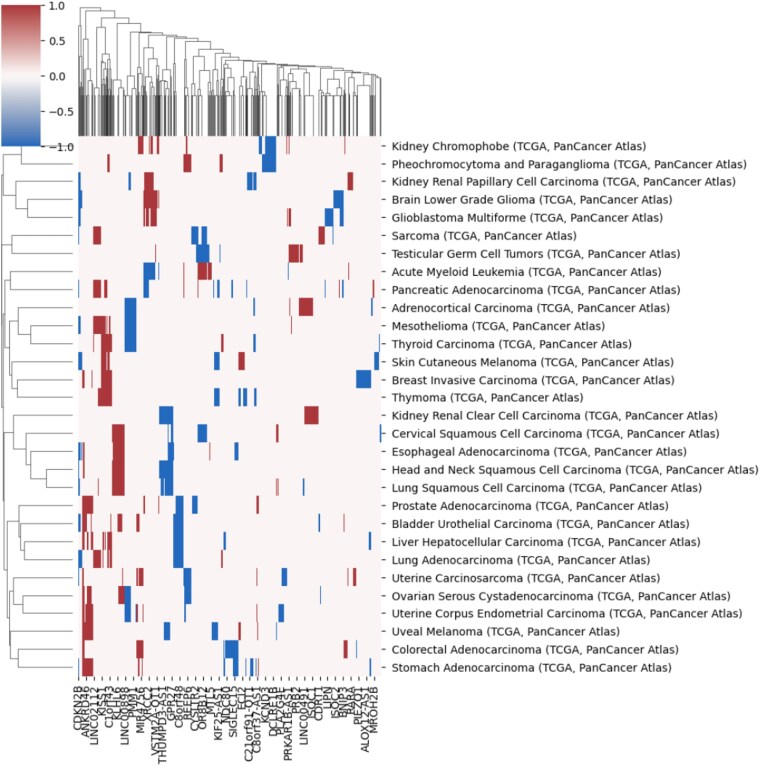
Clustering heatmap of CNA signatures across 30 cancer types. The columns represent normalized average CNV intensities for selected genes. Blue and red indicate duplications and deletions, respectively.

**Figure 5:**
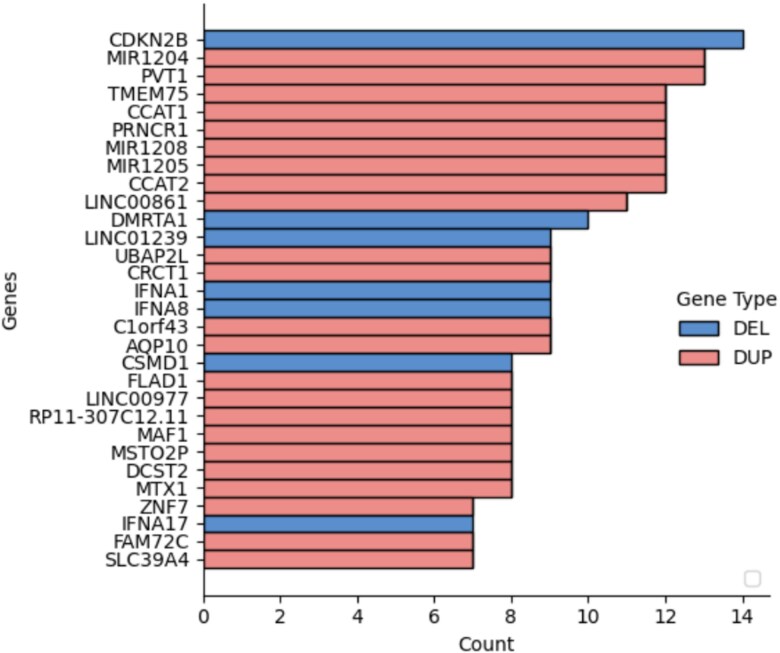
Top 30 most frequent signature genes across cancer types.

Using the procedures described above, we generated a set of feature genes for each cancer type from the CNA data. These features were used to construct abstracted CNA profiles that preserve only the most discriminative alterations per sample. These signatures simplify complex CNA patterns into 1008 informative genes. A Random Forest model trained on these features achieved comparable accuracy with the full dataset, confirming that the selected genes retain the essential classification signal. Figure 4 illustrates a clustering heatmap of signatures, showing that cancers with shared tissue origins often group together. Figure 5 shows the most frequently selected genes; notably, duplications were more common than deletions, consistent with oncogene activation. CDKN2B deletion was the most recurrent CNA across all cancer types. To assess biological relevance, we performed GO enrichment analysis on the signature genes. The results showed significant associations with cancer-related processes, including tumor suppressor activity and pathways annotated as ”HTLV-1 infection,” which likely reflect shared immune and signaling components rather than direct viral mechanisms.

In the majority of copy number studies, analyses of tumor samples are focused on identifying the driver genes or the focal regions. The genes in the signatures rather reflect the uniqueness of each sample or each cancer type. It is important to note that the feature genes are not intended as the only nor the optimal representation. The fundamental objective of the study is to explore the potential driver mutations that are infrequent but relevant to specific cancer types. Although the signatures do not imply pathogenic causation, we can instead reveal their potential implications and correlations by investigating the signatures and the feature genes. In general, spatial and annotation analysis suggest that some feature genes reflect structural and functional alternations in samples; the signatures of different cancer types show high preference in several genomic regions that suffer frequent CNAs in many cancer types.

### Signature comparison

We further compared the identified signatures with two related studies: Steele *et al*. [33] and Nguyen *et al*. [34] (Table 1). CNAttention achieved strong consistency with both arm-level and gene-level CNA frameworks. Arm-level comparison with CNAs dependent on the size of TCGA (Nguyen *et al*.) showed a mean Spearman $\rho $ of 0.28 (up to 0.48 in PAAD, GBM, and OV), recapitulating canonical gains of 8q and losses of 9p/10q. Gene-level overlap with Steele *et al*. yielded a median Jaccard of 0.008, with the highest concordance for CN4 and CN10–14 components, which correspond to 7p/8q gains and 17p/18q losses. Across all studies, $\sim $200 genes were shared among CNAttention, Steele, and TCGA, including key drivers (MYC, TP53, CDKN2A, PTEN). Notably, 60% of CNAttention genes were unique and enriched in immune and metabolic pathways, suggesting that the attention-based model captures finer, cancer-specific CNA patterns beyond existing pan-cancer signatures.

**Table 2:** Quantitative comparison of **CNAttention** with Steele *et al*. [33] and Nguyen *et al*. [34]

Metric	CNAttention (This study)	Steele *et al*. [33]	Nguyen *et al*. [34]	Interpretation
Arm-level concordance	**Median $\rho $=0.28** (max 0.48 in PAAD)	–	Median arm CNA freq $\approx $ 0.30 across cancers	Consistent arm-level CNA patterns (8q gain, 9p/10q loss).
Gene-level overlap	**Median J=0.008** (0.004–0.013)	21 CN signatures (explaining $\sim $85% variance)	–	Similar gene regions (7p/8q, 17p/18q alterations).
Shared genes (3-way)	** $\sim $ 200 (20%)**	$\sim $ 1800 genes in CN signatures	$\sim $ 3000 genes on recurrent arms	Core CNA drivers: *MYC*, *TP53*, *CDKN2A*, *PTEN*.
Unique genes	** $\sim $ 600 (60%)**	–	–	Novel immune- and metabolism-related CNAs.

### Validating copy number aberration signatures with a large-scale copy number variation reference database

**Figure 6:**
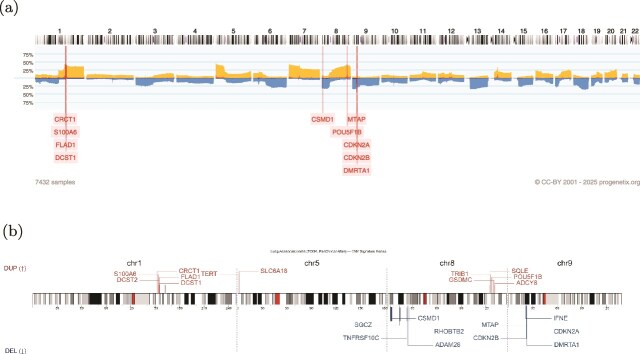
Comparison between external LUAD CNA frequency (Progenetix) and CNAttention-derived gene signatures. The highlighted regions on chr1, chr5, chr8, and chr9 correspond to recurrent CNAs (e.g. MYC amplification and CDKN2A deletion) that are consistent with external LUAD frequency data, demonstrating that CNAttention captures known recurrent CNAs. (a) Genome-wide CNA frequency profile of LUAD from the Progenetix database. The orange (above center line; up) and blue (below center line; down) bars represent copy-number gains and losses, respectively, with the y-axis showing alteration frequency across 7422 samples. (b) CNAttention-derived LUAD gene signatures projected onto chromosomes. The red and blue bars denote genes associated with duplication and deletion signatures, respectively, and the y-axis indicates their importance scores.

**Figure 7:**
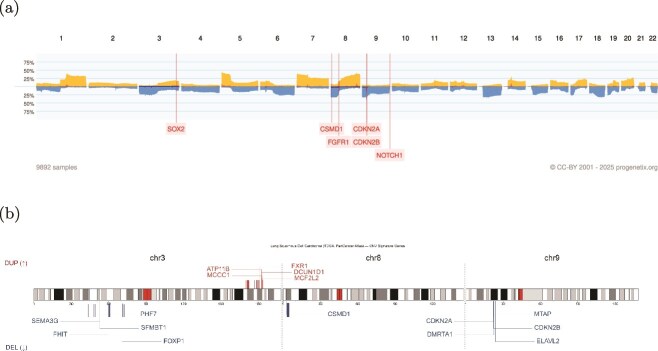
Comparison between the CNA frequency profile and CNV signature genes of LUSC. (a) CNA frequency plot of LUSC from the Progenetix database (see legend of Fig. 6 for format). The red dashed boxes highlight recurrently altered regions containing key oncogenes or tumor suppressors. (b) CNV signature gene plot of LUSC derived from the TCGA Pan-Cancer dataset. The red and blue bars indicate amplification- and deletion-associated signature genes, respectively. Chromosome ideograms are shown along the horizontal axis. The relative genomic positions of each gene are labeled, illustrating that the identified CNV signature genes largely correspond to regions of frequent CNAs observed in the Progenetix cohort.

We further extend our signatures to external datasets from Progenetix, which includes more heterogeneous CNV profiles from multiple data sources, to evaluate whether our signatures can extract the CNV pattern of certain cancer types. Here are the examples of lung adenocarcinoma and lung squamous cell carcinoma (Figs 6 and 7).

Lung adenocarcinoma (LUAD) and Lung squamous cell carcinoma (LUSC) represent two major histological subtypes of lung cancer, each characterized by distinct molecular profiles, including unique patterns of CNAs. In LUAD it has been shown previously that clonal loss of functional TP53 is significantly associated with subclonal gains of MCL-1 (1q21.2) [35]. Distinct CNA patterns characterize LUSC, with frequent amplifications of genes such as SOX2 (SRY-Box Transcription Factor 2) on chromosome 3q26 and FGFR1 (Fibroblast Growth Factor Receptor 1) on chromosome 8p11.23 driving oncogenic signaling pathways essential for cell proliferation and survival. Deletions affecting genes like NOTCH1 (Neurogenic Locus Notch Homolog Protein 1) on chromosome 9q34.3 are commonly observed, leading to dysregulated signaling cascades and accelerated tumor progression. In addition, loss of tumor suppressor genes CDKN2A/2B and CSMD1 are shared between LUAD and LUSC signatures. In conclusion, signatures can extract the features with relevance to the specific cancer, and also keep the common CNA pattern between LUAD and LUSC, help uncovering the relationships between different cancers.

### Similarities of neural crest originated subtypes

**Figure 8:**
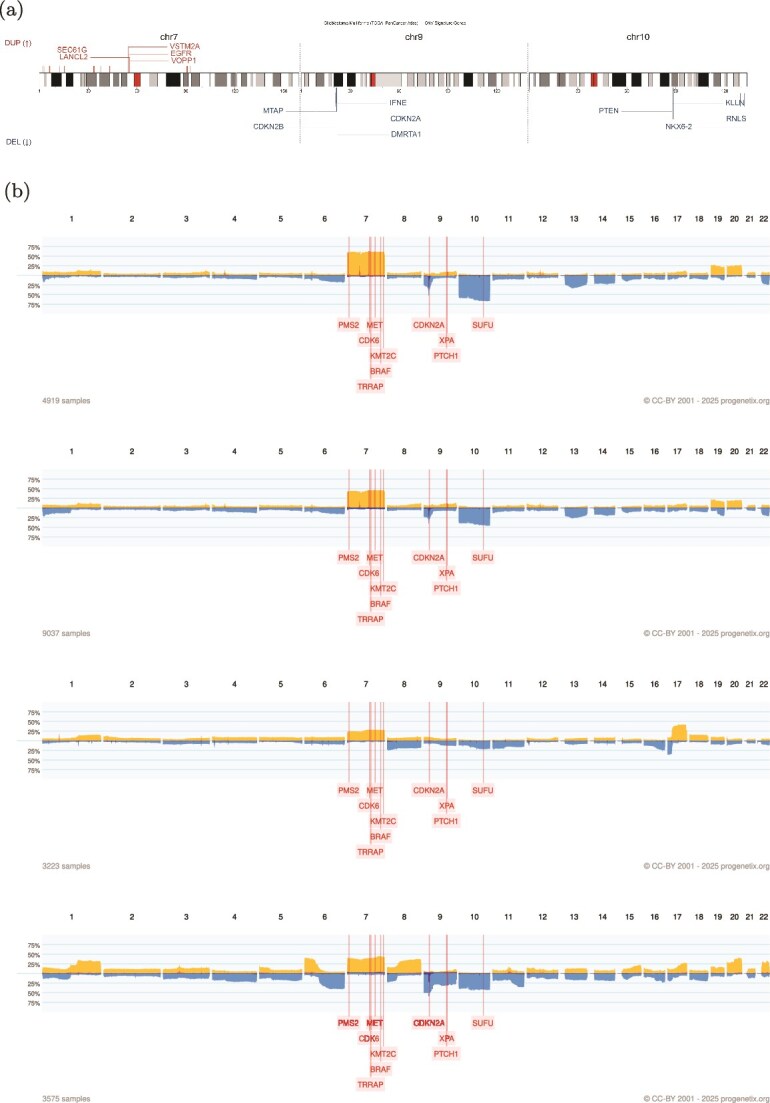
Comparison between the glioblastoma CNV gene signature and CNV frequency profiles of neural crest–derived tumor subtypes. Panel (a) presents the glioblastoma CNV gene signature from TCGA PanCancer Atlas. Panel (b) shows vertically aligned genome-wide CNV frequency plots for glioblastoma, glioma, medulloblastoma, and melanoma from the Progenetix database, highlighting shared amplification and deletion landscapes across these related tumors. (a) CNV gene signature of glioblastoma showing recurrent amplification (above chromosome, red) and deletion (below, blue) genes along chromosomal locations. (b) Genome-wide CNV frequency plots of glioblastoma, glioma, medulloblastoma, and melanoma from the Progenetix database.

We further extend the signatures to more external datasets, by importing the cancer classification tree in Progenetix, and we find the similarities of neural crest-originated subtypes, including glioblastoma, glioma, medulloblastoma, and melanoma. The four distant cancer types exhibit highly similar signatures in both feature selection and alteration frequencies. Figure 8 illustrates the comparison of original CNA data, features, and known drivers of these cancers on chromosomes with similar signatures. Notably, their signatures show high similarities in the duplication of chromosome 7 and the deletion of chromosomes 9 and 10. Additionally, they share pairwise similarities in the duplication of chromosome 1 and 20, as well as the deletion of chromosome 14.

Chromosome 7, with frequent copy number gains in all four cancers, harbors several key oncogenes such as EGFR, CDK6, and MET in glioma; KMT2C and PMS2 in medulloblastoma; BRAF, RAC1, and TRRAP in melanoma. Similarly, chromosome 9 and 10, commonly deleted in these cancers, contain several important suppressor genes such as CDKN2A and PTEN in glioma; XPA, PPP6C, and CDKNA in melanoma; PTCH1 and SUFU in medulloblastoma. Notably, the CDKN2A/B deletion is the most frequent CNA across all cancer types.

In the 1990s, epidemiological studies [36] initially uncovered a link between melanoma and nervous system tumors. This association was not only observed in familial cases, confirmed by germline mutations, but also indicated a significantly elevated risk of one disease in individuals with a history of the other. Despite evidence suggesting shared pathophysiological pathways and responsiveness to similar drugs, the genetic connection between these two disease groups remained largely elusive [37].

Despite their clinical and histological disparities, medulloblastomas, melanomas, and gliomas all originate from neural crest cell lineages. Recent research on neural crest cells and cancers derived from these lineages suggests that malignant cells mimic various aspects of neural crest development at a behavioral, molecular, and morphological level [38]. Aberrations in tumor cells may reactivate embryonic developmental programs, thereby promoting tumorigenesis and metastasis. In melanoma, WNT family members, crucial during the epithelial-to-mesenchymal transition of neural crest cells, are reactivated during invasive transformation [39]. In glioblastoma, experimental data suggest dysregulated WNT signaling supports the onset of cancer stem cells, facilitating tumor enlargement and metastasis [39]. In medulloblastoma, the WNT subgroup represents one of the four molecular subtypes of the disease [40]. Regions with similar signatures show abnormal amplification frequencies in several WNT genes, potentially reflecting overexpression of WNT signaling. Specifically, WNT2B and WNT4 exhibit moderate amplification frequencies, while WNT2, WNT3A, WNT9A, and WNT16 demonstrate high amplification frequencies. WNT2 and WNT16 are signature genes in all three subtypes, indicating their prevalence among individual samples.

### Copy number variation heterogeneity reflects cancer subtypes

**Figure 9:**
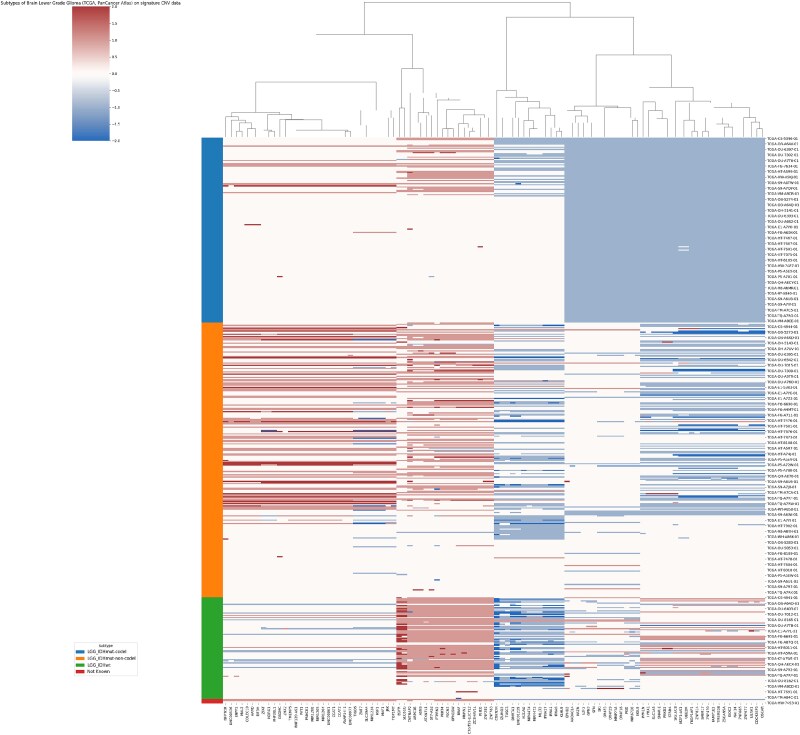
Subtypes of brain LGG on signature CNV data. The different subtypes (separated by different colors on the left) show three different CNA patterns.

To find out whether these signatures reflect the heterogeneity within cancers, we collected all available clinical information and adopted a random forest to see whether the signatures could help classify the subtypes. The results show that for all cancers, compared with using all CNV profiles, using signatures can help increase the accuracy, indicating that signatures can reflect the heterogeneity of subtypes within cancer. Here we showcase how signature reflects subtypes of brain lower grade glioma (LGG) in Fig. 9 by the 1p/19q co-deletion.

## Discussion

In this study, we compiled an extensive collection of cancer CNA profiles to pinpoint genomic aberration signatures specific to each cancer type. Our novel attention-based MIL method, CNAttention, showcased the potential of CNA patterns in tumor identification. Focusing on isolating unique components in diagnostic CNA profiles, we extracted signatures for 30 cancer types, each characterized by a minimal gene representation with high discrimination capacity.

Comparative analyses of signature genes and their respective regions revealed frequent duplications on chromosomes 7 and 8, and deletions on chromosome 22 across various cancer types. Despite their prevalence, these regions also exhibited features with high differentiating power, possibly indicating the functional significance of cancer-related genes specific to pathway involvement.

Our analyses unveiled shared CNA signatures among four clinically and pathologically distinct cancer types—glioblastoma, medulloblastoma, melanoma, and glioma. These tumor types can be traced back developmentally to common lineages of neural crest cells. Although research has sporadically linked neural crest cells with glioma and melanoma development, the genetic underpinnings of their association in oncogenetic processes remain elusive. Through comparative analysis of shared mutations and improvements in developmental processes in normal tissues, insights into shared pathologies and potential therapeutic targets may emerge. In addition, the signatures reveal the heterogeneity of cancer types, and shed light on uncovering more potential cancer subtypes.

In summary, this study presents a systematic pipeline for integrative and comparative analyses of a large amount of copy number data. The resulting CNA signatures offer new perspectives on the understanding of common foundations in cancers and show promising potential in applications of tumor classification.

**Key Points**

- We present CNAttention, an attention-based multiple-instance learning framework for pan-cancer classification using somatic copy number aberrations (CNAs).
- CNAttention achieves robust performance across diverse tumour types in TCGA PanCancer Atlas data, outperforming classical machine-learning baselines and recent deep-learning models.
- The attention mechanism identifies representative samples and highlights informative genomic regions, enabling interpretable cancer-typespecific CNA signatures at both chromosome-arm and gene levels.
- Comparative analyses with recent pan-cancer CNA studies show that CNAttention recovers known driver alterations while revealing additional immune- and metabolism-related signatures.

## Supplementary Material

CNAttention_supplementary_bbaf696

## Data Availability

The gene-level somatic copy number aberrations analysed in this study were obtained from publicly available TCGA PanCancer Atlas cohorts via the cBioPortal for Cancer Genomics (https://www.cbioportal.org). External datasets for CNA signatures validation are from (https://www.progenetix.org) under the corresponding studies. Processed CNA matrices and the CNAttention code used to generate the results in this manuscript are available at https://github.com/baudisgroup/CNAttention.
