# Supplementary material for: CNAttention: an attention-based deep multiple-instance method for uncovering copy number aberration signatures across cancers
Source: Brief Bioinform. 2026 Jan 15;27(1):bbaf696. doi: 10.1093/bib/bbaf696 (PMC12805253; doi:10.1093/bib/bbaf696)
Supplement: CNAttention_supplementary_bbaf696 [file cnattention_supplementary_bbaf696.pdf]

Supplementary Material for CNAttention: an attention-based deep multiple-instance method for uncovering CNA signatures across cancers

Ziying Yang[1,2] and Michael Baudis[1,2*]

[1*]Department of Molecular Life Sciences, University of Zurich, Winterthurerstr. 190, Zurich, 8057, Zurich, Switzerland.
[2]Swiss Institute of Bioinformatics, Zurich, Switzerland.

*Corresponding author(s). E-mail(s): michael.baudis@mls.uzh.ch;
Contributing authors: ziying.yang@uzh.ch;

S1 Methodology

S1.1 Feature selection by RFECV

RFECV, which stands for Recursive Feature Elimination with Cross-Validation, is a feature selection technique commonly used in machine learning for identifying the most relevant features in a dataset. The RFECV algorithm works by recursively removing features from the dataset and evaluating the performance of the model using cross-validation at each step. It starts with all features and iteratively removes the least important ones based on a specified criterion (e.g., model accuracy or another performance metric).

The RFECV algorithm can be summarized as follows:

$$\text{RFECV}(X, y, \text{est}, \text{scoring}) = \\ \underset{\text{selected features}}{\text{argmax}} (\text{mean}(\text{cross\_val\_score}(\text{est}, X_{\text{selected features}}, y, \text{scoring}))) \tag{1}$$

where:

- X is the feature matrix,

- y is the target vector,
- estimator is the machine learning model used for evaluation,
- scoring is the evaluation metric (e.g., accuracy, precision, recall).

S1.2 Cancer classification by CNAttention

In typical machine learning problems like image classification, it is assumed that an image clearly represents a category (a class). However, in many real-life applications, multiple instances are observed and only a general statement of the category is given. This scenario is called multiple instance learning (MIL [1, 2]). MIL deals with a bag of instances for which a single class label is assigned. Hence, the main goal of MIL is to learn a model that predicts a bag label, e.g., a medical diagnosis. In our scenario of cancer classification by CNA profiles, since the heterogeneity of cancers, CNA profiles of the same cancer may look different, and we are more focused on the general CNA pattern of cancers. Therefore, we treat each sample as an instance, to ensure that when representative samples of a specific class are in the same bag, this bag is more likely to show a general pattern in this cancer type. In this way, the general pattern of cancers instead of individual CNA profiles are learned. An additional challenge is to discover key instances [3], *i.e.* the instances that trigger the bag label; i.e., the representative CNA patterns of a certain class. By uncovering the key instances, we will be able to reveal the general CNA patterns in the cancer landscape as well as CNA heterogeneity within cancers, which may be related to cancer subtypes. In this paper, we propose a new method CNAttention that extends [4] to multiple classification scenarios of cancer classification based on CNA profiles. We formulate the MIL model using the Bernoulli distribution for the bag label and train it by optimizing the log-likelihood function. We show that the application of the Fundamental Theorem of Symmetric Functions provides a general procedure for modeling the bag label probability (the bag score function) that consists of three steps: (i) a transformation of instances to a low-dimensional embedding, (ii) a permutation-invariant (symmetric) aggregation function, and (iii) a final transformation to the bag probability. We parameterize all transformations using neural networks (i.e., a combination of convolutional and fully-connected layers), which increases the flexibility of the approach and allows us to train the model in an end to-end manner by optimizing an unconstrained objective function. In addition, we use a trainable weighted average where weights are given by a two-layered neural network. The two-layered neural network corresponds to the attention mechanism [5, 6]. Notably, the attention weights allow us to find key instances.

Problem formulation In the classical (binary) supervised learning problem, the objective is to find a model that predicts a binary value for a target variable, $y \in 0, 1$, given an instance $\mathbf{x} \in \mathbb{R}^D$. In the case of the MIL problem, however, instead of a single instance, there is a bag of instances, $X = \mathbf{x}_1, \ldots, \mathbf{x}_K$, which exhibit neither dependency nor ordering among each other. It is assumed that K could vary for different bags. In our scenario, assume that there are N classes (i.e., cancer types), and each instance is assigned a label from 1 to N, representing its class or category. The bag label (Y) represents the distribution of class labels within the bag. For each class $i \in 1, ..., N$, $Y[i]$ indicates the proportion of instances in the bag belonging to class i. This distribution captures the diversity of class labels within the bag. Furthermore,

we assume that individual labels exist for the instances within a bag, i.e., $y_1, \ldots, y_K$, where $y_k \in 1, ..., N$ for $k = 1, \ldots, K$, however, these labels are not accessible during training and remain unknown. We can express the assumptions of the MIL problem in a concise form using the maximum operator:

$$Y = \max_k y_k. \tag{2}$$

These assumptions indicate that a MIL model must be permutation-invariant. However, optimizing an objective based on the maximum over-instance labels could present challenges, especially in the context of multiple classifications. Gradient-based learning methods may encounter issues such as vanishing gradients, and this formulation is applicable only when employing an instance-level classifier.

To address these challenges inherent in multiple classifications, we train a MIL model by optimizing the log-likelihood function. In this approach, the bag label distribution follows a multinomial distribution, where each class has a probability $\theta_i(X) \in [0, 1]$ of being present in the bag of instances X. Here, $\theta_i(X)$ represents the probability of class i given the bag of instances X.

S1.3 MIL with Neural Networks

In classical MIL problems, it is typically assumed that instances are represented by features that do not require further processing, i.e., f serves as the identity function. However, for certain tasks such as image or text analysis, additional feature extraction steps may be necessary. Moreover, theorems in [7] suggest that for a sufficiently flexible class of functions, any permutation-invariant score function can be modeled. Therefore, we consider a class of transformations parameterized by neural networks $f_\psi(\cdot)$ with parameters ψ, which transform the k-th instance into a low-dimensional embedding, $hk = f\psi(x_k)$, where $h_k \in \mathcal{H}$ and $\mathcal{H} = [0, 1]$ for the instance-based approach, and $\mathcal{H} = \mathbb{R}^M$ for the embedding-based approach.

Ultimately, the parameter $\theta(X)$ is determined by a transformation $g_\phi : \mathcal{H}^K \rightarrow [0, 1]$. In the instance-based approach, the transformation g_ϕ is simply the identity function, while in the embedding-based approach, it could also be parameterized by a neural network with parameters ϕ.

The concept of parameterizing all transformations using neural networks is highly attractive, as it allows for arbitrary flexibility in the approach and enables end-to-end training via backpropagation. The only requirement is that the MIL pooling operation must be differentiable.

S1.4 Attention-based MIL pooling

Attention Mechanism: We employ a weighted average of instances (low-dimensional embeddings), where the weights are determined by a neural network. Additionally, these weights must sum to 1 to maintain invariance to the bag size. The weighted average satisfies the conditions of theorem [7], where the weights, along with the embeddings, constitute part of the f function. Let $H = h1, \ldots, hK$ denote a bag of K embeddings. We apply the following MIL pooling mechanism:

$$z = \sum_{k=1}^{K} a_k h_k, \tag{3}$$

where the attention weights a_k are calculated as follows:

$$a_k = \frac{\exp w^\top \tanh\left(Vhk^\top\right)}{\sum j = 1^K \exp w^\top \tanh\left(Vh_j^\top\right)}, \tag{4}$$

where $w \in \mathbb{R}^{L\times 1}$ and $V \in \mathbb{R}^{L\times M}$ are parameters. Additionally, we employ the hyperbolic tangent $\tanh(\cdot)$ element-wise non-linearity to allow for both negative and positive values, facilitating proper gradient flow. This proposed construction enables the discovery of (dis)similarities among instances.

Gated Attention Mechanism: We applied augmenting the $\tanh(\cdot)$ non-linearity with a gating mechanism, yielding the following formulation:

$$a_k = \frac{\exp\{w^\top\big(\tanh\left(Vh_k^\top\right) \odot \mathrm{sigm}\big(Uh_k^\top\big)\big)\}}{\sum_{j=1}^{K} \exp\{w^\top\big(\tanh\left(Vh_j^\top\right) \odot \mathrm{sigm}\big(Uh_j^\top\big)\big)\}} \tag{5}$$

where $w \in \mathcal{R}^{L\times 1}$,$V \in \mathcal{R}^{L\times M}$, $U \in \mathcal{R}^{L\times M}$ are learnable parameters, $\odot$ is an element-wise multiplication and $sigm()$ is the sigmoid non-linearity. The gating mechanism introduces a learnable non-linearity that potentially removes the troublesome linearity in $tanh()$.

The attention-based MIL pooling allows for assigning varying weights to instances within a bag, enabling the bag-level classifier to discern key instances and generate highly informative bag representations. Moreover, coupling the attention-based MIL pooling with transformations f and g parameterized by neural networks renders the entire model fully differentiable and adaptive. These characteristics render the proposed MIL pooling mechanism highly flexible, capable of modeling an arbitrary permutation-invariant score function.

Ideally, for a bag with the label ($Y = i$), high attention weights should be assigned to instances likely to have label $y_k = i$ (key instances). Thus, the attention mechanism facilitates easy interpretation of the decision in terms of instance-level labels. While the attention network does not directly provide scores like the instance-based classifier, it can be considered a proxy for it. The attention-based MIL pooling bridges the gap between the instance-level and embedding-level approaches.

S1.5 Signature generating

After applying MIL, we still need to transfer the bag label to the instance label, therefore transferring the instance weights to feature weights for each unique class. Also, we need the instance label for evaluating our method. To calculate the instance accuracy, we first need to obtain the weighted predictions for each instance. This can be achieved by multiplying the attention weights with the class predictions for each instance within each bag and accumulating these weighted predictions across bags.

Let $w_{i,j}$ represent the attention weight for instance i in bag j, and $p_{i,k}$ denote the class prediction for class k for instance i. The weighted prediction $wp_{i,k}$ for class k and instance i is computed as follows:

$$wp_{i,k} = w_{i,j} \times p_{i,k}$$

After obtaining the weighted predictions for each instance, we normalize these predictions by dividing them by the sum of attention weights for each instance across all bags. Let n_i represent the normalization factor for instance i, which is the sum of attention weights for instance i across all bags. The normalized prediction $\hat{p}_{i,k}$ for class k and instance i is computed as follows:

$$\hat{p}_{i,k} = \frac{wp_{i,k}}{n_i}$$

Once we have the normalized predictions for each instance, we can determine the predicted class for each instance by selecting the class with the highest normalized prediction. Let $\hat{c}_i$ represent the predicted class for instance i, which is obtained by:

$$\hat{c}_i = \arg\max_k \hat{p}_{i,k}$$

Finally, we compare the predicted classes with the ground truth labels for each instance to compute the instance accuracy, defined as the proportion of instances for which the predicted class matches the ground truth label.

The instance accuracy can be calculated using the following formula:

$$\text{Accuracy} = \frac{\text{Number of correctly predicted instances}}{\text{Total number of instances}}$$

S2 CNA signatures

By using the data processing and modeling procedures described in the Methodology section, we generated a panel of feature genes for each cancer type from the collected CNA samples. Then, with the combination of feature genes of each cancer type, we create an abstract representation for each copy number profile, where only alternations that contributed to the distinctiveness of the sample were preserved. Fig. S1 compares the original CNA patterns with the derived signature features where the frequent and extensive regional alterations in the original data have been replaced by 1008 feature genes, which visibly compare to subsets of characteristic changes in the original CNA data and represent the most discriminative alternations.

Table S1: The number of samples and cancer subtypes in the studied cancers.

**Cancer Type**	**Sample Number**
Acute Myeloid Leukemia	191
Adrenocortical Carcinoma	89
Bladder Urothelial Carcinoma	408
Brain Lower Grade Glioma	511
Breast Invasive Carcinoma	1070
Cervical Squamous Cell Carcinoma	293
Colorectal Adenocarcinoma	592
Esophageal Adenocarcinoma	182
Glioblastoma Multiforme	575
Head and Neck Squamous Cell Carcinoma	517
Kidney Chromophobe	65
Kidney Renal Clear Cell Carcinoma	509
Kidney Renal Papillary Cell Carcinoma	283
Liver Hepatocellular Carcinoma	367
Lung Adenocarcinoma	511
Lung Squamous Cell Carcinoma	487
Mesothelioma	87
Ovarian Serous Cystadenocarcinoma	572
Pancreatic Adenocarcinoma	183
Pheochromocytoma and Paraganglioma	161
Prostate Adenocarcinoma	489
Sarcoma	253
Skin Cutaneous Melanoma	367
Stomach Adenocarcinoma	438
Testicular Germ Cell Tumors	149
Thymoma	123
Thyroid Carcinoma	497
Uterine Carcinosarcoma	56
Uterine Corpus Endometrial Carcinoma	523
Uveal Melanoma	80

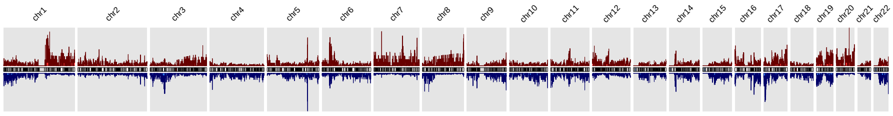

(a) Original CNAs.

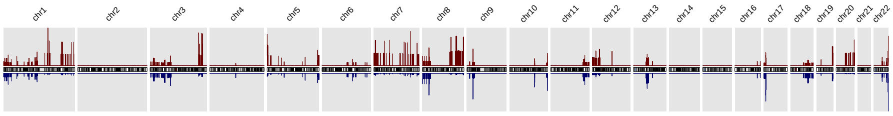

(b) CNA signatures.

Fig. S1: The copy number alternation landscape of all samples using the original CNAs and the feature genes. The feature genes are able to dramatically reduce the complexity of CNA signals while maintaining the mutational characteristics. The red colors above the chromosome axis represent the average amplifications, and the blue colors below the chromosome axis represent the average deletions.

References

[1] Dietterich, T.G., Lathrop, R.H., Lozano-Pérez, T.: Solving the multiple instance problem with axis-parallel rectangles. Artificial intelligence **89**(1-2), 31–71 (1997)

[2] Maron, O., Lozano-Pérez, T.: A framework for multiple-instance learning. Advances in neural information processing systems **10** (1997)

[3] Liu, G., Wu, J., Zhou, Z.-H.: Key instance detection in multi-instance learning. In: Asian Conference on Machine Learning, pp. 253–268 (2012). PMLR

[4] Ilse, M., Tomczak, J., Welling, M.: Attention-based deep multiple instance learning. In: International Conference on Machine Learning, pp. 2127–2136 (2018). PMLR

[5] Bahdanau, D., Cho, K., Bengio, Y.: Neural machine translation by jointly learning to align and translate. arXiv preprint arXiv:1409.0473 (2014)

[6] Raffel, C., Ellis, D.P.: Feed-forward networks with attention can solve some long-term memory problems. arXiv preprint arXiv:1512.08756 (2015)

[7] Zaheer, M., Kottur, S., Ravanbakhsh, S., Poczos, B., Salakhutdinov, R.R., Smola, A.J.: Deep sets. Advances in neural information processing systems **30** (2017)
